# High alcohol consumption and early hip fracture risk in men and women

**DOI:** 10.1038/s41598-026-43095-6

**Published:** 2026-03-14

**Authors:** Charlotta Elleby, Pia Skott, Sven-Erik Johansson, Sven Nyrén, Holger Theobald, Helena Salminen

**Affiliations:** 1 Department of Dental Medicine, Karolinska Institutet, Huddinge, Sweden; 2 Academic Centre for Geriatric Dentistry, Stockholm, Sweden; 3 Public Dental Services, Folktandvården, Stockholm, Sweden; 4 Centre for Primary Health Care Research, Department of Clinical Sciences, Lund University, Malmö, Sweden; 5 Department of Radiology, Karolinska University Hospital, Solna, Sweden; 6 Division of Family Medicine and Primary Care, Department of Neurobiology, Care Sciences and Society, Karolinska Institutet, Huddinge, Sweden; 7 Academic Primary Care Health Centre, Region Stockholm, Stockholm, Sweden

**Keywords:** Fracture risk assessment, Hip fractures, Osteoporosis, High alcohol consumption, Alcoholism, Epidemiology, Fracture repair, Orthopaedics, Risk factors, Musculoskeletal system

## Abstract

Our aim was to study whether hospitalizations due to high alcohol consumption, as an objective indicator of high alcohol consumption, is associated with hip fractures in non-elderly adults. The participants in this study consist of 10,043 men and women in a survey study in Stockholm 1969-70, when they were 18–25 years old. We used data on hospitalization events due to a hip fracture or a diagnosis indicating high alcohol consumption from 1970 to 2016 acquired from the National Patient Register. This allowed 47 years of follow-up, until the participants were 65–72 years old, an age-span that can be described as “non-elderly adults”. A Cox regression model was used to estimate the hazard ratio (HR) of hip fractures, comparing individuals with and without a diagnosis indicating high alcohol consumption, treating this exposure as a time-varying covariate. There were 151 participants with at least one hip fracture, 86 women and 65 men. There were 450 participants, 173 women and 277 men, who had an alcohol related event, as an indication of alcohol abuse, during the follow-up time of which 24 (10 women and 14 men) also experienced a hip fracture. We found an elevated HR for hip fractures for non-elderly women (HR = 4.59, 95% CI = 2.12–9.95, *p* < 0.001) and men (HR = 7.65, 95% CI = 4.07–14.36, *p* < 0.001) with a previous hospitalization-diagnosis indicating high alcohol consumption.

**Supplementary Information:**

The online version contains supplementary material available at 10.1038/s41598-026-43095-6.

## Introduction

 Fragility fractures are common in Sweden: about 50% of Swedish women and 25% of Swedish men are expected to have one during their lifetime^1^. Fragility fractures are fractures of the hip, upper arm, wrist, and vertebrae, caused by low energy trauma. Hip fractures have the most serious consequences such as loss of independency and even death^2,3^. The mean age for a fragility fracture in Sweden is high: for a hip fracture it is about 84 years in women and 79 years in men^4^. Osteoporosis and risk of falling are two of the factors that contribute to the risk of fragility fractures, and alcohol can affect both. The action of alcohol on bone tissue can be both direct and indirect, with more bone resorption and less bone formation, leading to less bone mass^5^. Therefore, we expect an association between high alcohol consumption and hip fractures. Some studies do show that an ongoing or recent high alcohol consumption can lead to osteoporosis and affect the risk of falling in older persons^6,7^. However, when studying alcohol consumption and risk of hip fractures, results are not unanimous.

In a cohort of Swedish females, 50–81 years old, Baron et al. found no association between high alcohol consumption and risk of hip fractures^8^, and neither did Johnell et al. when studying Mediterranean women > 50 years^9^. A study of a Norwegian cohort found that the risk of hip fracture for men aged 30–59 years with a high alcohol consumption was up to three times higher than that for moderate drinkers, but there were no significant results for women or older men^10^. A meta-analysis of alcohol consumption and hip fractures from 2015, including 18 studies with a total of 3.7 million participants of various ages and both genders, found that heavy alcohol consumption was associated with a higher risk of sustaining hip fractures than for non-drinkers, with a pooled relative risk (RR) of 1.71 (CI 1.42–2.01)^11^. A more recent systematic review came to a similar conclusion with a significantly elevated risk for hip fracture^12^. Another systematic review and meta-analysis showed a significant positive association between alcohol consumption and overall fracture risk (RR 1.35, CI 1.01–1.81) but not with hip fractures (RR 1.19, CI 0.96–1.48)^13^. However, they found that sex, duration of follow-up, mean age of participants and adjusting for smoking, BMI etc. can influence the results, which could explain the failure to find an association between high alcohol consumption and hip fractures in this meta-analysis.

The age of the population is important when studying alcohol consumption and hip fracture risk. Of all hip fractures, 2–11% occur in the non-elderly^14^, a definition generally including adults under 65 years, but the upper limit can vary from 60 to 75 years. In a study of young (20–49 years old) and middle aged (50–69 years old) Swedish men and women with hip fractures whose alcohol consumption was assessed using the Alcohol Use Disorder Identification Test (AUDIT)^15^, high alcohol consumption was twice as common (26%) as in the general population (13%) in both age-groups with no gender difference^16^. In a Danish-Swedish study of women and men with hip fractures under 60 years old, also using AUDIT to assess alcohol consumption, harmful consumption was found in 25% of the women and 31% of the men^17^. AUDIT is a validated tool for assessment of alcohol consumption but only reflects the situation during one year prior to and at the time of the assessment, and it is not an objective measure. Consumption of large amounts of alcohol during an extended period, is most likely to affect the risk factors for fragility fractures. Therefore, studies have been made with more objective measures. Karantana et al. studied women in the UK over and under 65 years old who had sustained a hip fracture and found that 18% of the women in the younger age group had a history of alcohol abuse using objective data such as medical records^18^. In a study of 86 middle-aged Swedish men, 50–64 years old, with a hip fracture, one third were found to have a history of alcohol dependence found in objective data^19^. In another study of an English/Danish population, with and without alcoholic cirrhosis, the hazard ratio (HR) for hip fracture was 17.9 (CI 7.8–40.8)/16.4 (CI 11.4–23.6) for those who were < 45 years old with the disease compared with those without, while the HR was only 2.1 (CI 1.1–2.2)/3.0 (2.1–4.2) in those > 75 years old^20^. Pasoto et al. studied hip fractures in a Brazilian cohort of non-elderly (18–64 years) as well as elderly (> 64 years) men and women and found that in the younger age-group, 38.5% had a previous history of alcoholism in their medical records, while this was true only for 4.7% in the older age-group^21^. Conclusively, although results of studies of the association of alcohol consumption and hip fractures at large are diverging, studies indicate that high alcohol consumption is more common among non-elderly men and women with hip fractures. However, to our knowledge, there are no studies of hip fracture risk prediction in non-elderly with non-self-reported high alcohol consumption.

## Aims

Our aim was to investigate whether hospitalizations with a diagnosis code indicating high alcohol consumption, as an objective measure of high alcohol consumption, was associated with hip fractures in non-elderly adults.

## Materials and methods

### Study design

This is a cohort study with a follow-up period of up to 47 years, including participants from the REBUS cohort, where REBUS is short for Rehabiliteringsbehovsundersökningen (which is translated to “the Assessment of Needs of Rehabilitation”), and data from the Swedish National Patient Register (NPR) kept by the Swedish National Board of Health and Welfare.

### Cohort

The REBUS cohort consisted of a selected random sample of 32,183 inhabitants of the approximately 450,000 inhabitants of the County of Stockholm, 18–65 years old, who in 1969–1970 received a postal questionnaire to assess the medical and social situation of the adult population, and their met and unmet needs for service and rehabilitation^22^. The response rate was 88%. Our study include the responders who turned 18–25 years in 1970 and they were followed until they were 65–72 years old. We omitted the participants who were deceased, had a hip fracture or alcohol diagnosis during 1969, resulting in a sample of 10,043 participants, 5,390 women and 4,653 men.

### Data

**Fig. 1:**
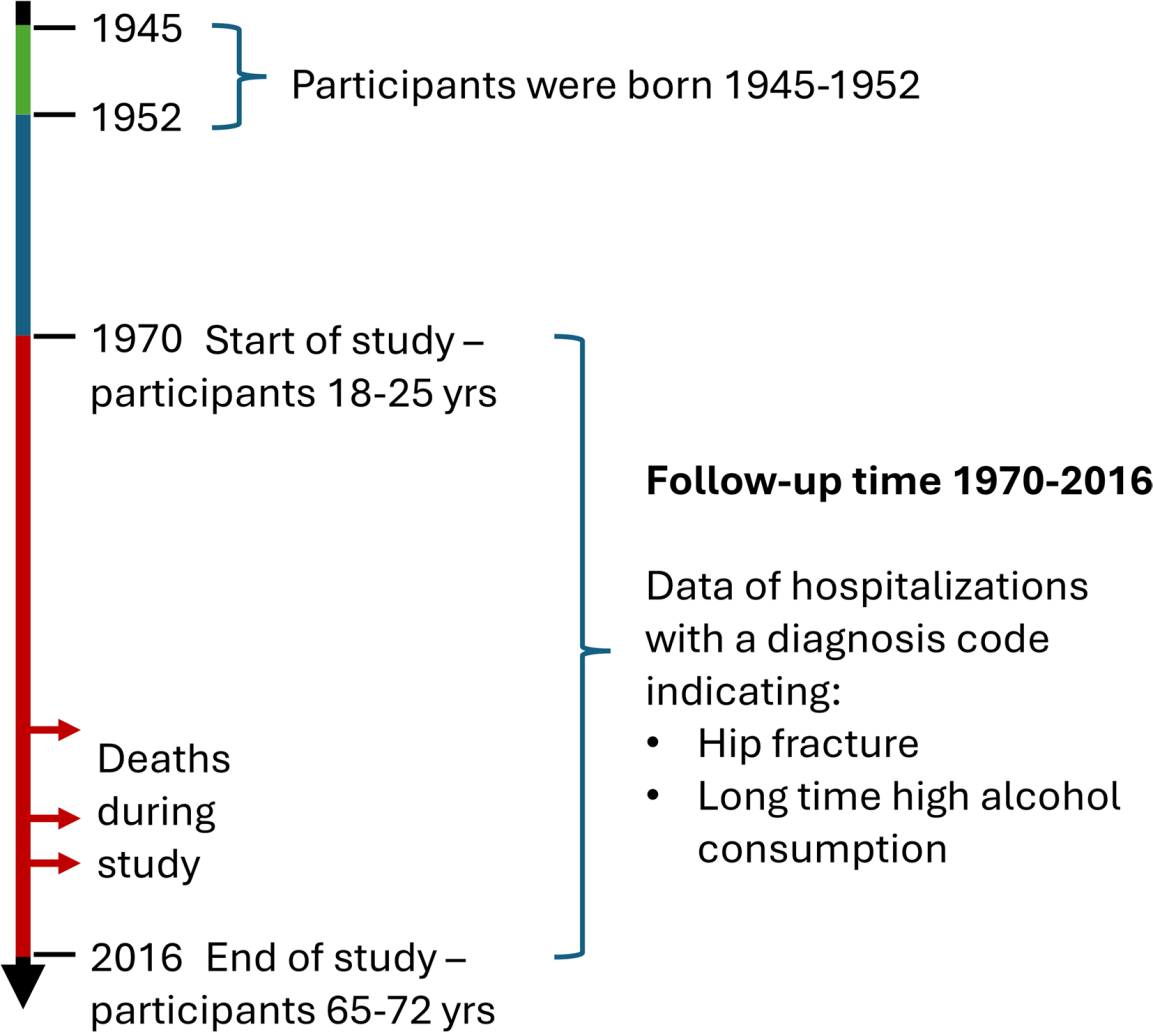
Timeline of the study.

We acquired patient data from the NPR 1 December 2017 on hospital admissions due to hip fractures from 1 January 1970 to the end of the study, 31 December 2016, enabling up to 47 years of follow-up time (Fig. 1). The only fragility fracture we used was fracture of the hip since they always require hospitalization and are seldom undetected. The diagnosis codes used were S720, S721, S722, S723, S72 according to ICD10 for events after 1996. The codes 820, 820 A, 820B, 820 C, 820D, 820 W, 820X, 821, 821 A, 821B according to ICD9 were used for events between 1 January 1987 and 31 December 1996, and codes 820, 821, 8200, 8201, 8210, 8211 according to ICD8 for events 1 January 1970–31 December 1986.

We defined “hip fracture” as fractures of the femoral neck and peri- and sub-trochanteric fractures. The different ICD systems do not match exactly, and location of the fracture was sometimes incomplete in the NPR data. Therefore, we chose to include rather than omit so as not to miss any fractures. We had no records of what kind of trauma preceded the fractures but had checked this in a sample of this cohort and found that the vast majority was low energy trauma^23^. Excluding high energy trauma may underestimate the prevalence of fragility fractures^24^ and we are advised by Cummings and Eastell not to make any distinction between fractures resulting from high or low energy trauma^25^. We therefore included all hip fractures, but only the first one since we could not separate new fractures from complications of a previous fracture. Date of death of the participants who were deceased at the end of the study was acquired from the Swedish Cause of Death Register, which is also kept by the Swedish National Board of Health and Welfare.

We acquired diagnosis codes for hospitalizations specified to be due to high alcohol consumption, either as a main diagnosis or as a secondary diagnosis, during the whole follow-up time. The following ICD-10 diagnosis codes were used for events 1 January 1997 -31 December 2016: F10, G31.2, G62.1, G72.1, I42.6, K29.2, K85.2, K70, T51.0, Y90.7, Y90.8, Y91.2 and Y91.3. For events 1 January 1987–31 December 1996, we used the corresponding codes according to ICD-9: 291, 303, 305 A, 425 F, 535D, 571 A-D, 790D and 980. For events 1 January 1970–31 December 1986, we used the codes according to ICD-8: 291, 303, 5710 and 5711. The diagnoses include mental and behavioural disorders due to use of alcohol, degeneration of the nervous system due to alcohol, alcoholic polyneuropathy, alcoholic myopathy, alcoholic cardiomyopathy, alcoholic gastritis, alcohol induced acute pancreatitis, alcoholic liver disease, toxic effect of alcohol, alcohol levels in blood ≥ 200 mg/ml, severe and very severe alcohol intoxication. As well as for the hip fracture data, we chose to include rather than omit not to miss any events if the codes did not correspond exactly, and we also only included the first hospitalization event due to high alcohol consumption. The diagnoses we included indicate a long-term high alcohol consumption, or abuse.

### Statistical analyses

The statistical analyses were performed using Stata Statistical Software, version Stata/IC 14.2 for Windows^26^. The significance level was set at p-values ≤ 0.05. We used a Cox regression model with age at the start of the study included in the model. We analysed hazard ratio of hip fractures separately for men and women, with and without an alcohol-related diagnosis before the fracture, adjusting for age at start of the study. The exposure of interest, i.e. having an alcohol-related diagnosis, was treated as a time-varying covariate. The assumption of proportional hazard ratio was tested using Schoenfeld residuals.

### Ethical approval 

This study was approved by the Regional Ethical Review Board Stockholm on 22 June 2016 (Registration number 2016/902-31/2) and was, as well as the original REBUS study, performed according to the 1964 Helsinki declaration and its later amendments. The REBUS participants gave their written informed consent at the start of the study in 1969-1970. At that time, there was only an advisory ethical committee at Karolinska Institutet and there was no formal ethical application. However, the committee was aware of the study and did not object to it. Several follow-up studies have since been approved and in 1996, the local ethics committee at Karolinska Institutet required that the participants be informed that their data were to be used for studies then and in the future and that they had the right to decline. This was done satisfactorily through advertisements in national papers. The local ethics committee and, after 2004, the Regional Ethical Review Board, have since then considered the participants to have given their consent to future studies. The digitalized data of the participants, including personal identity numbers and the answers from the postal surveys, were sent to the Swedish National Board of Health and Welfare for the addition of fracture and alcohol related hospitalization data. The returned data were anonymized and contained no personal identity numbers so the researchers could not identify individual participants.

## Results 

The participants were followed from the start of the study, 1 January 1970, until the end of the study, 31 December 2016, during which period events of hospitalizations with diagnosis codes indicating a high alcohol consumption and codes for hip fractures, and deaths were registered. The median age at study start was 22 years (Interquartile range 22–23 years) for both men and women.

There were 450 participants with an event of hospitalization with a diagnosis code indicating a high alcohol consumption during the 47 years of follow-up (Table 1). The event was more common in men than in women and the median age was lower for men than for women. There were 151 individuals with at least one hip fracture, but no significant difference between men and women. (Table 1). We found that 24 individuals, 10 women and 14 men, had both a hip fracture and an alcohol diagnosis. In 19 of these 24 cases (80%), the hip fracture occurred after the alcohol diagnosis.

**Fig. 2:**
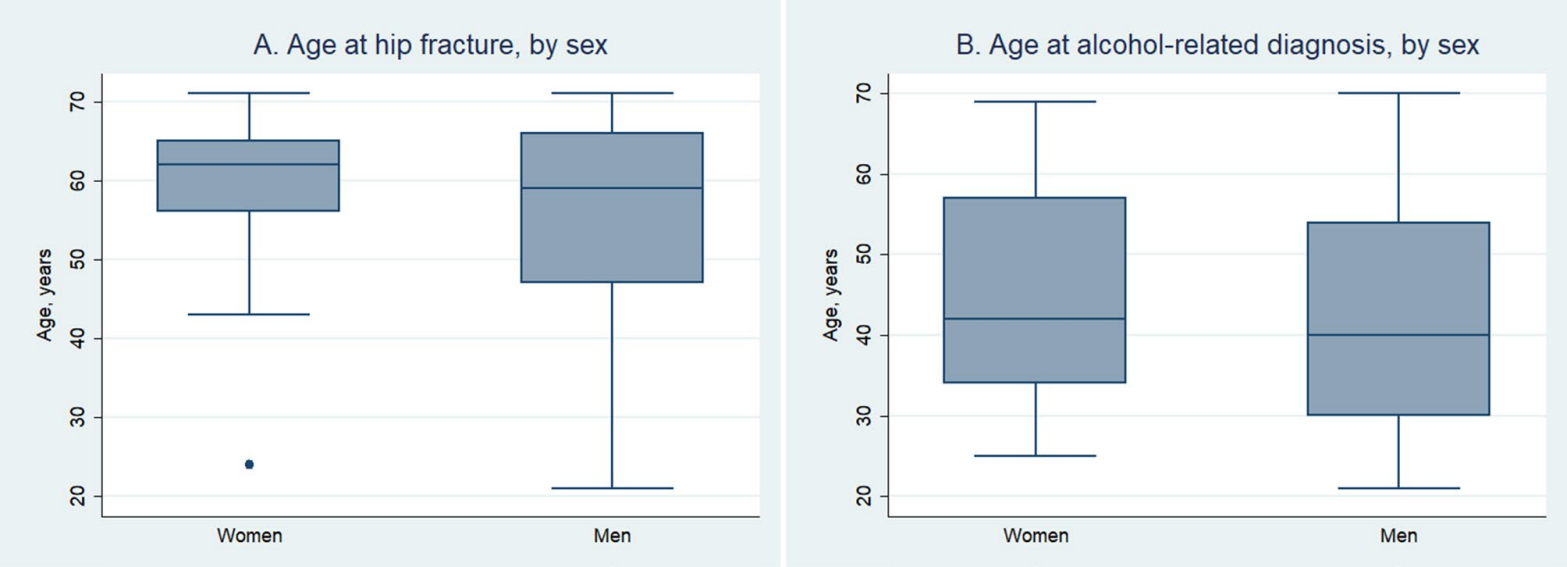
(**a**) Age at hip fracture and (**b**) Age at hospitalization with a diagnosis code indicating high alcohol consumption.

The distribution of hip fractures and alcohol diagnoses by age and sex is shown in Fig. 2.

We calculated the hazard ratio of hip fracture, comparing person-time with and without an alcohol related diagnosis, treating alcohol related diagnosis as a time-varying exposure. We analyzed men and women separately and adjusted for age at study-start. (Table 2)

**Table 1:** Description of cohort by sex and totals.

	Total	Women	Men	*p*-value sex-difference
Total number of participants, *n* (%)	10,043 (100)	5,390 (53.7)	4,653 (43.3)	
Median age at study-start, years (Interquartile Range, IQR)	22 (22–23)	22 (22–23)	22 (22–23)	0.912 ^b^
Deceased at end of study, n (%)	1,299 (12.9)	573 (10.6)	726 (15.6)	< 0.001^a^
No events (hip fracture or death) n (%)	8,635 (86.0)	4,758 (88.3)	3,877 (83.3)	< 0.001^a^
Participants with ≥ 1 hip fracture, n (%)	151 (1.5)	86 (1.6)	65 (1.4)	0.415^a^
Median age at hip fracture, years (95% CI)	61 (59–63)	62 (60–63)	59 (53–63)	0.0623^b^
Number of person years in total	453,537	245,736	207,802	
Mean duration of the follow-up, years, (95% CI)	45.2 (45.0-45.3)	45.6 (45.5–45.7)	44.7 (44.5–44.9)	< 0.001^b^
Participants with an alcohol diagnosis, n (%)	450 (4.5)	173 (3.2)	277 (6.0)	< 0.001^a^
Median age at alcohol diagnosis, years (95% CI)	41 (39–43)	42 (39–45)	40 (38–43)	0.0445^b^
Participants with both a hip fracture *and* an alcohol diagnosis, n	24	10	14	0,238^a^
Alcohol diagnosis before the fracture, n (%)	19 (80)	7 (70)	12 (86)	0,615^c^
Alcohol diagnosis after the fracture, n (%)	5 (21)	3 (30)	2 (14)	

a= Chi^2^-test, b= Wilcoxon rank sum test, c= Fisher’s exact test.

**Table 2:** Cox regression, proportional hazard ratio (HR) with 95% confidence interval (95% CI) of hip fracture with alcohol diagnosis as a time varying exposure adjusted for age at study start.

Hip fracture				
	Women		Men	
	HR	95% CI	HR	95% CI
Alcohol related diagnosis				
No (reference)	1	-	1	-
Yes	4.59	2.12–9.95	7.65	4.07–14.36

Using the Cox model, we found significantly higher hazard ratios (HR) for hip fractures for both women (HR = 4.59; 95% CI = 2.12–9.95, *p* < 0.001) and men (HR 7.65, 95% CI 4.07–14.36, *p* < 0.001) for those with an alcohol diagnosis before a fracture. We tested proportional HR using Schoenfeld residuals with the result for women: alcohol diagnosis: χ²(1)= 2.7, p=0.101, start-age: χ²(1)=0.66, p=0.417, Global test χ²(2)=3.34, p=0.188, and for men: alcohol diagnosis: χ²(1)=1.59, p=0.207, start-age: χ²(1)=0.12, p=0.727, Global test χ²(2)=1.71, p=0.426, and the assumption of proportional HR was thereby found to be fulfilled.

## Discussion

We found that high alcohol consumption, identified by diagnosis codes at events of hospitalization, was associated with a substantially increased risk of sustaining a hip fracture for non-elderly women (HR 4.59, 95% CI 2.12–9.95) and non-elderly men (HR 7.65, 95% CI 4.07–14.36), using a Cox regression model. Even though women had more hip fractures than men in our study, the higher HR for hip fractures for men than women indicate that the influence of alcohol on the risk of fracture is higher for men than for women.

The HR for hip fractures in our study was higher than in other studies using self-reported data of alcohol consumption^10,27–29^, but within the same range as the study by Otete et al., where they used hospital data of liver cirrhosis as a sign of high alcohol consumption, thus including only the heaviest drinkers^20^. Previous studies have shown that objective measures of high alcohol consumption, such as medical records of alcoholism, alcohol dependence, alcohol abuse, and alcohol cirrhosis, are more common in non-elderly with a hip fracture than in those without a fracture^18,19,21,30^. Our way of defining high alcohol consumption includes all these diagnoses, with the addition of diagnoses that indicate long-time high alcohol consumption. The use of these diagnoses, with their objective character and most likely only including those with the heaviest alcohol consumption, could be one explanation why the HR is higher in our study (4.59 for women and 7.65 for men) compared with others that use self-reported measures of alcohol consumption (HR 1.2–3.2)^10,27–29^.

Another explanation to our study presenting higher hazard ratios for hip fractures than other studies, may be that we only include hip fractures that occur relatively early in life, until the participants were 65–72 years old. Yuan et al. made a similar study using hospital data indicating high alcohol consumption resulting in a HR of 2.56 for hip fracture, but they only included participants who were 65 years and older at study start^31^.

Hospital diagnoses indicating a high alcohol consumption were more common in men than in women in our cohort, which is in line with previous findings. Normally, women have a higher incidence of fragility fractures than men when including all ages^1^. By restricting the analysis to non-elderly individuals and thereby excluding hip fractures occurring at the most common age (around 80 years), age-related osteoporosis becomes less influential. Other risk factors, such as high alcohol consumption, may then exert a greater impact on fracture risk.

The age distribution of hip fractures is not normally distributed during the 47 years of the study. As the population gets older, the hip fractures become more common, with a median age of 61 years (Table 1; Fig. 2). This is more pronounced in women which could mean that some of the hip fractures that men sustain relatively early in life could result from accidents and high energy trauma. Men would be expected to participate in more activities with risk of accidents than women. However, omitting fractures caused by high energy trauma is not advised as previously explained^24,25^. The age distribution of alcohol related events is more evenly distributed with a median age of 41 years (Fig. 2).

This study contains 47 years of data. This is a long period, in which there have been significant changes in healthcare practices, diagnostic criteria, and social behaviours related to alcohol consumption. This adds some uncertainty to our results. The patient attrition and missing data could also be a problem, especially during such a long period. The diagnosis data is national, but we may have lost track of the participants due to emigration, for example. The study population consists of persons who lived in Stockholm in 1969–1970 and may not be representative of other populations. Differences in healthcare systems, cultural attitudes towards alcohol, and genetic factors could limit the generalizability of the findings. A weakness in our study is also that we have no records of smoking habits. Smoking has a negative effect on bone mineral density and has been found to be an important risk for hip fractures in the non-elderly^17,18^. Smoking is more common among consumers of high amounts of alcohol^32^ and could potentially be a confounding factor. Since we rely only on hospitalizations with diagnose codes indicating a long-term high alcohol consumption to identify those with a high alcohol consumption, we miss those who have not been hospitalized which could lead to an underestimation of the true prevalence of high alcohol consumption. This, in turn, would mean that our regression model may have underestimated the hazard ratios. 

The participants in this study were born in 1945–1952. Swedish women in this age-group had a higher consumption of alcohol than previous generations of women during the study, diminishing, but not eradicating, the sex-difference in drinking pattern^33,34^.

The alcohol related diagnoses and the diagnoses for hip fractures are not exact, and the translation between the different ICD-systems is not a perfect match, so this implies some uncertainty. In some cases, the ICD codes have become more specific with each version. For instance, to identify high alcohol consumption, we used four codes according to ICD-8, eight in ICD-9 and 16 codes in ICD-10. This means that the specificity in diagnosing high alcohol consumption is not constant during the time of the study.

A strength in our study is the objective measure of alcohol consumption since self-reported alcohol consumption is not a reliable source of information and often underestimated, which could affect the results. We also have an almost complete register of occurred hip fractures in our dataset, as the Swedish system of personal identity numbers enables registrations of diagnosis-data of hospitalizations. This was introduced before the start of the study and was completely established in all Stockholm hospitals in 1972. Including most of the adult non-elderly life of the participants with our long study period of 47 years is also a strength.

The variable “high alcohol consumption” is difficult to study. There is no defined level of “high consumption”, and self-reported alcohol consumption may be underestimated due to its sensitive nature. Our findings contribute to establishing the relationship between high alcohol consumption and fracture risk. However, since alcohol consumption is such a difficult variable to define and to measure, bias corrected studies are warranted to confirm these associations.

## Conclusion

We found that events of hospitalization with a diagnose code indicating a long-term high alcohol consumption was associated with 4.6 times and 7.7 times increased hazard of sustaining a hip fracture for non-elderly women and men respectively.

## Supplementary Information

Below is the link to the electronic supplementary material.

Supplementary Material 1

## Data Availability

All data generated or analysed during this study are included in this published article [and its supplementary information files].

## References

[1] Kanis, J. A. et al. Long-term risk of osteoporotic fracture in Malmo. Osteoporosis international: a journal established as result of cooperation between the European Foundation for Osteoporosis and the National Osteoporosis Foundation of the USA. **11**(8) 669–674. (2000). 10.1007/s001980070064 11095169

[2] Rohde, G., Haugeberg, G., Mengshoel, A. M., Moum, T. & Wahl, A. K. Two-year changes in quality of life in elderly patients with low-energy hip fractures. A case-control study. *BMC Musculoskelet. Disord.* **11**, 226 (2010). 20920239 10.1186/1471-2474-11-226 PMC2954991

[3] Johnell, O. et al. Mortality after osteoporotic fractures. Osteoporosis international: a journal established as result of cooperation between the European Foundation for Osteoporosis and the National Osteoporosis Foundation of the USA.**15**(1) 38–42. (2004). 10.1007/s00198-003-1490-4 14593451

[4] Mattisson, L., Bojan, A. & Enocson, A. Epidemiology, treatment and mortality of trochanteric and subtrochanteric hip fractures: data from the Swedish fracture register. *BMC Musculoskelet. Disord.* **19** (1), 369 (2018). 30314495 10.1186/s12891-018-2276-3 PMC6186067

[5] Abukhadir, S. S., Mohamed, N. & Mohamed, N. Pathogenesis of alcohol-induced osteoporosis and its treatment: a review. *Curr. Drug Targets*. **14** (13), 1601–1610 (2013). 24138635 10.2174/13894501113146660231

[6] Pasco, J. A. et al. High Alcohol Intake in Older Men and the Probability of Osteoporotic Fracture According to the FRAX Algorithm. *Nutrients* ;**13**(9). (2021). 10.3390/nu13092955 PMC8468672 34578830

[7] Du, F. et al. Association of osteoporotic fracture with smoking, alcohol consumption, tea consumption and exercise among Chinese nonagenarians/centenarians. *J. Nutr. Health Aging*. **15** (5), 327–331 (2011). 21528157 10.1007/s12603-010-0270-z PMC12880287

[8] Baron, J. A. et al. Cigarette smoking, alcohol consumption, and risk of hip fracture in women. *Arch. Intern. Med.* **161** (7), 983–988 (2001). 11295961 10.1001/archinte.161.7.983

[9] Johnell, O. et al. Risk factors for hip fracture in European women: the MEDOS Study. Mediterranean Osteoporosis Study. *J. bone mineral. research: official J. Am. Soc. Bone Mineral. Res.* **10** (11), 1802–1815 (1995). 10.1002/jbmr.5650101125 8592959

[10] Søgaard, A. J. et al. The association between alcohol consumption and risk of hip fracture differs by age and gender in Cohort of Norway: a NOREPOS study. *Osteoporos. international: J. established as result cooperation between Eur. Foundation Osteoporos. Natl. Osteoporos. Foundation USA*. **29** (11), 2457–2467 (2018). 10.1007/s00198-018-4627-1 30006884

[11] Zhang, X., Yu, Z., Yu, M. & Qu, X. Alcohol consumption and hip fracture risk. *Osteoporos. international: J. established as result cooperation between Eur. Foundation Osteoporos. Natl. Osteoporos. Foundation USA*. **26** (2), 531–542 (2015). 10.1007/s00198-014-2879-y 25266483

[12] Ke, Y. et al. Alcohol consumption and risk of fractures: a systematic review and dose-response meta-analysis of prospective cohort studies. *Adv. Nutr.* (2023). 10.1016/j.advnut.2023.03.008 PMC10334160 36966875

[13] Asoudeh, F., Salari-Moghaddam, A., Larijani, B. & Esmaillzadeh, A. A systematic review and meta-analysis of prospective cohort studies on the association between alcohol intake and risk of fracture. *Crit. Rev. Food Sci. Nutr.* **62** (20), 5623–5637 (2022). 33596741 10.1080/10408398.2021.1888691

[14] Rogmark, C. et al. Hip fractures in the non-elderly-Who, why and whither? *Injury* **49** (8), 1445–1450 (2018). 29983171 10.1016/j.injury.2018.06.028

[15] Saunders, J. B., Aasland, O. G., Babor, T. F., de la Fuente, J. R. & Grant, M. development of the alcohol use disorders identification test (AUDIT): WHO collaborative project on early detection of persons with harmful alcohol consumption–II. *Addiction* **88** (6), 791–804 (1993). 8329970 10.1111/j.1360-0443.1993.tb02093.x

[16] Al-Ani, A. N. et al. Risk factors for osteoporosis are common in young and middle-aged patients with femoral neck fractures regardless of trauma mechanism. *Acta Orthop.* **84** (1), 54–59 (2013). 23343373 10.3109/17453674.2013.765639 PMC3584603

[17] Strøm Rönnquist, S. et al. Frailty and osteoporosis in patients with hip fractures under the age of 60-a prospective cohort of 218 individuals. Osteoporosis international: a journal established as result of cooperation between the European Foundation for Osteoporosis and the National Osteoporosis Foundation of the USA. ;**33**(5):1037–1055. (2022). 10.1007/s00198-021-06281-y PMC9007814 35029719

[18] Karantana, A. et al. Epidemiology and outcome of fracture of the hip in women aged 65 years and under: a cohort study. *J. Bone Joint Surg. Br.* **93** (5), 658–664 (2011). 21511933 10.1302/0301-620X.93B5.24536

[19] Jónsson, B., Sernbo, I., Kristensson, H. & Johnell, O. Hip fractures in middle-aged men: a consequence of early retirement and alcohol misuse? *Alcohol Alcohol*. **28** (6), 709–714 (1993). 8147979

[20] Otete, H. et al. Hip fracture risk in patients with alcoholic cirrhosis: A population-based study using English and Danish data. *J. Hepatol.* **69** (3), 697–704 (2018). 29673756 10.1016/j.jhep.2018.04.002

[21] Pasoto, S. G. et al. Osteoporotic hip fractures in non-elderly patients: relevance of associated co-morbidities. *Rheumatol. Int.* **32** (10), 3149–3153 (2012). 21947377 10.1007/s00296-011-2154-x

[22] Bygren, L. O. Met and unmet needs for medical and social services. *Scandinavian J. social Med. Suppl.* **8**, 1–134 (1974). 4533362

[23] Elleby, C. et al. Two methods of evaluating mandibular trabecular pattern in intraoral radiographs and the association to fragility fractures during a 47-year follow up. *Eur. J. Oral. Sci.* **129** (5), e12801 (2021). 34101266 10.1111/eos.12801

[24] Sanders, K. M. et al. The exclusion of high trauma fractures may underestimate the prevalence of bone fragility fractures in the community: the Geelong Osteoporosis Study. *J. bone mineral. research: official J. Am. Soc. Bone Mineral. Res.* **13** (8), 1337–1342 (1998). 10.1359/jbmr.1998.13.8.1337 9718203

[25] Cummings, S. R. & Eastell, R. *Stop (mis)classifying fractures as high- or low-trauma or as fragility fractures. Osteoporosis international: a journal established as result of cooperation between the European* (Foundation for Osteoporosis and the National Osteoporosis Foundation of the USA, 2020). 10.1007/s00198-020-05325-z 32173783

[26] StataCorp. Available from: https://www.stata.com

[27] Wang, S. M. et al. Association of alcohol intake and fracture risk in elderly varied by affected bones: a nationwide longitudinal study. *Psychiatry Investig*. **17** (10), 1013–1020 (2020). 33059395 10.30773/pi.2020.0143 PMC7596281

[28] Mukamal, K. J., Robbins, J. A., Cauley, J. A., Kern, L. M. & Siscovick, D. S. Alcohol consumption, bone density, and hip fracture among older adults: the cardiovascular health study. Osteoporosis international: a journal established as result of cooperation between the European Foundation for Osteoporosis and the National Osteoporosis Foundation of the USA. ;**18**(5):593–602. (2007). 10.1007/s00198-006-0287-7 17318666

[29] Prieto-Alhambra, D. et al. Smoking and alcohol intake but not muscle strength in young men increase fracture risk at middle age: a cohort study linked to the swedish national patient registry. *J. bone mineral. research: official J. Am. Soc. Bone Mineral. Res.* **35** (3), 498–504 (2020). 10.1002/jbmr.3917 31714618

[30] Høidrup, S., Grønbaek, M., Gottschau, A., Lauritzen, J. B. & Schroll, M. Alcohol intake, beverage preference, and risk of hip fracture in men and women. copenhagen centre for prospective population studies. *Am. J. Epidemiol.* **149** (11), 993–1001 (1999). 10355374 10.1093/oxfordjournals.aje.a009760

[31] Yuan, Z. et al. Effects of alcohol-related disease on hip fracture and mortality: a retrospective cohort study of hospitalized Medicare beneficiaries. *Am. J. Public. Health*. **91** (7), 1089–1093 (2001). 11441736 10.2105/ajph.91.7.1089 PMC1446699

[32] Garnett, C., Oldham, M., Shahab, L., Tattan-Birch, H. & Cox, S. Characterising smoking and smoking cessation attempts by risk of alcohol dependence: A representative, cross-sectional study of adults in England between 2014–2021. *Lancet Reg. Health Eur.* **18**, 100418 (2022). 35814338 10.1016/j.lanepe.2022.100418 PMC9257647

[33] Bengtsson, C. et al. Alcohol habits in Swedish women: Observations from the population study of women in Gothenburg, Sweden 1968–1993. *Alcohol Alcohol. (Oxf. Oxfs.)*. **33**, 533–540 (1998). 10.1093/alcalc/33.5.533 9811207

[34] Raninen, J. & Agahi, N. Trends in older people’s drinking habits, Sweden 2004–2017. *Nordisk Alkohol Nark*. **37** (5), 459–469 (2020). 35310773 10.1177/1455072520954336 PMC8899064

